# A bacteriophage-based virus-like particle vaccine induces cross-reactive neutralising antibodies against porcine epidemic diarrhoea viruses (PEDV)

**DOI:** 10.1186/s13567-025-01559-z

**Published:** 2025-07-01

**Authors:** Jixiang Gu, Xu Zheng, Chunhui Li, Shipeng Wang, Xiangyu Xie, Martin F. Bachmann, Yuchen Nan, Liang Li, Pei Sun, Lisha Zha, Xinyue Chang

**Affiliations:** 1 College of Veterinary Medicine, Anhui Agricultural University, Hefei, Anhui China; 2 Department of Preventive Veterinary Medicine, College of Veterinary Medicine, Northwest Agriculture and Forestry University, Yangling, Shanxi China; 3 Department of Biomedical Research, University of Bern, Bern, Switzerland; 4 Shenzhen Herz Life Science and Technology Co., Ltd, Shenzhen, China

**Keywords:** PEDV, AP205-VLP, vaccine, neutralizing antibody

## Abstract

Although vaccines against porcine epidemic diarrhoea viruses (PEDV) are available, PED outbreaks continue to occur in many countries due to the emergence of new variants. Therefore, further endeavours are necessary to develop efficient and broadly protective vaccines. In this context, we present a nanoparticle vaccine candidate, referred to as AP205-S1, which successfully elicited antibody responses in mice and pigs. The vaccine was created by coupling the S1 protein of PEDV-KB2013, a G-II strain, to a bacterially expressed AP205-VLP using the SpyCatcher/SpyTag system. The AP205-S1 vaccine demonstrated an intact and homogenous viral particle structure, incorporating *E. coli*-derived ssRNA. Upon administration in mice, AP205-S1 induced high levels of S1-specific IgG antibodies in both serum and the gastrointestinal tract, particularly following a booster dose. Importantly, these antibodies were capable of neutralising PEDV in vitro, suggesting that the vaccine can generate protective antibodies against PEDV infection. Notably, the antibodies elicited by AP205-S1 exhibited cross-neutralising potential against a G-I strain, PEDV-AH2018-HF1, which was preserved in our lab. Additionally, S1-specific IgG antibodies were stimulated in piglets following immunisation with AP205-S1, and these antibodies could neutralise PEDV in vitro. Interestingly, piglets immunised with AP205-S1 exhibited lower viral loads compared to control piglets following a viral challenge. In conclusion, we developed a VLP-based vaccine candidate against PEDV, which demonstrated excellent immunogenicity in both mice and piglets, potentially providing protection against viral infection. Our work offers an effective option for preventing future PEDV epidemics.

**Supplementary Information:**

The online version contains supplementary material available at 10.1186/s13567-025-01559-z.

## Introduction

Since porcine epidemic diarrhoea viruses (PEDVs) were first reported in the 1970s, they have caused significant losses to the global pork industry [1, 2]. PEDV is an ssRNA virus that belongs to the genus *Alphacoronavirus* within the *Coronaviridae* family. The genome of PEDV frequently mutates, leading to the continuous emergence of new variants [3, 4].

Based on the spike (S) protein sequence, PEDVs are categorised into two groups: classic strains (G-I) and mutant strains (G-II). Over the past decade, G-II strains have been predominant in the fields [5]. Notably, G-II strains are more pathogenic and contagious than G-I strains, resulting in higher mortality rates and numerous endemic outbreaks in different countries [1, 6].

Due to the significant genomic differences between G-I and G-II strains, the immune responses induced by G-I strains are poorly cross-reactive with G-II strains. As a result, the vaccines derived from G-I strains grant limited protection against G-II strains, thus hindering the eradication of the virus. The ideal approach is to develop a vaccine candidate that induces cross-protection against both G-I and G-II viruses.

PEDV primarily targets the gastrointestinal tracts of pigs, leading to symptoms such as vomiting, watery faeces, diarrhoea, and dehydration [7]. Upon entering the epithelial cells, the virus activates innate immune responses that involve dendritic cells, natural killer cells, and macrophages. These components then stimulate adaptive immunity through cytokine secretion and the processing of antigens [8, 9].

In response to these innate signals and viral antigens, B cells differentiate into plasma cells and secrete antigen-specific antibodies, serving as the predominant defence in hosts against PEDV invasion. Among these, IgG and IgA are the most prevalent antibody classes found in serum and mucosal tissues, respectively. They play crucial roles in defending hosts against PEDV infection [10]. Neutralising IgG antibodies are believed to effectively prevent PEDV from attaching to and entering host cells [11]. Additionally, high levels of IgA in serum have been associated with reduced faecal shedding of the virus.

Moreover, IgA antibodies present in colostrum and milk are vital for protecting suckling piglets against enteric viruses [12]. Recent studies have shown a strong correlation between neutralising antibody titres in the serum, faeces, and colostrum and S1 protein-specific IgA titers [13]. Therefore, an ideal vaccine for PEDV should aim to stimulate high levels of both IgG and IgA antibodies.

Although the specific porcine receptors for PEDV have not yet been identified, S protein plays a crucial role in the viral infection process. Independent studies have demonstrated that the S protein is an excellent target for subunit vaccine development [7, 14–16]. The 3D-structure of the PEDV S protein, revealed by Cryo-EM at a resolution of 3.1 Å, identifies the putative receptor binding domain (RBD) and sialic acid binding regions located within the S1 protein [17]. These regions are essential for the attachment of the virus to host cells and contain neutralising epitopes [18, 19].

Bacteriophage AP205-derived VLP, an icosahedral particle composed of 180 identical capsid proteins, has been utilized in various vaccine candidates. We previously fused the receptor binding motif (RBM) of SARS-CoV-2 to AP205, referring to it as AP205-RBM. This vaccine induced IgA production in mice following subcutaneous administration [20].

In addition to genetic fusion, chemical coupling using a linker is another established method to connect antigens with AP205-VLP. For instance, SMPH has been employed to link the RBD of SARS-CoV-2 to CuMV_TT_-VLP [21–23]. Moreover, a covalent bond formed between SpyCatcher and SpyTag (both bacterially derived sequences known to form covalent bonds) successfully presented the surface protein Pfs47 of *Plasmodium falciparum* on AP205 VLPs. This approach effectively induced specific high-affinity IgG antibodies in immunised mice [24].

The specific SpyCatcher/SpyTag covalent binding occurs spontaneously between lysine and aspartic acid under moderate conditions, and the production costs may be lower than those associated with chemical coupling. This presents a significant advantage for veterinary vaccines, particularly in the food-producing industry.

In this study, we developed a vaccine candidate for PEDV, AP205-S1, derived from the G-II virus PEDV-KB2013 using SpyCatcher/SpyTag linkage technology. AP205-S1 maintained the structure of intact viral particles and packaged *E. coli*-derived RNA during bacterial expression.

After immunising mice with AP205-S1, we observed the stimulation of systemic and mucosal IgG antibodies, with particularly high titres following a booster dose. Furthermore, sera from immunised mice exhibited significant PEDV-neutralising activity in vitro, as evidenced by a reduction in the cytopathogenic effect (CPE) caused by PEDV-KB2013 . Remarkably, some sera demonstrated 100% neutralising titres as high as 1:160 at day 49, indicating the strong ability of AP205-S1 to induce neutralising antibodies.

Additionally, the antibodies elicited by AP205-S1 not only recognised but also cross-neutralised a G-I strain, AH2018-HF1. In summary, AP205-S1 shows considerable promise for protecting pigs against PEDV infection and is worth pursuing for industrial application in the future.

## Materials and methods

### Expression and purification of AP205-SpyCatcher

The cDNA encoding the SpyCatcher domain was synthesised by General Biol (Anhui) Co. Ltd. This fragment was then ligated to pET28a-AP205 using LightNing DNA Assembly Mix Plus (BestEnzymes Biotech, Lianyungang, China). The resulting construct, AP205-SpyCatcher, was transformed into BL21 competent cells. To induce the expression of the AP205-SpyCatcher protein, 0.5 mM IPTG was added to the growth medium. After lysing the bacteria with sonication, the protein was precipitated with ammonium sulfate. Finally, the purified AP205-SpyCatcher was dialysed in a buffer containing 20 mM Tris–HCl. The protein was characterised by running SDS-PAGE gel, agarose gel, dynamic light scattering (DLS), and transmission electron microscope (TEM). 

### Production of S1-SpyTag

Vero cells were first infected with the PEDV-KB2013 strain. After infection, total RNA was extracted and reverse transcribed into cDNA. The sequence encoding the S1 protein was amplified from the cDNA using specific primers infused with the SpyTag-encoding sequence. This was then assembled into the pTWIST-CMV-BetaGlobin-WPRE-Neo vector (TWIST Bioscience, California, USA) under the control of the CMV promoter.

When the HEK293F cells reached a concentration of 1 × 10^6^/mL, the plasmid was incubated with PEI in a 1:3 ratio at room temperature for 15 min to form complexes, which were then added to the cell suspension. After five days, the cell supernatant containing the S1-SpyTag protein was collected. The target protein was purified using a Ni–NTA column (GenScript, Nanjing, China), followed by dialysis with PBS to remove imidazole. The purified protein was then concentrated and quantified using a BCA assay (Beyotime Biotech Inc., Jiangsu, China) before further application.

### Generation of the AP205-S1 vaccine

The purified AP205-SpyCatcher and S1-Spytag were connected by incubating at 4 °C for 16 h at a molar ratio of 1:1. After this incubation, the coupling product was loaded onto an SDS-PAGE gel, and the coupling efficiency was calculated based on densitometry analysis. The formed AP205-S1 was then loaded onto a 1% agarose gel to examine the packed RNA. Western blot using an AP205-specific antibody was then performed to verify the covalent bond.

### Transmission electron microscope (TEM)

VLPs at a 0.2 mg/mL concentration were dropped on a copper grid, which was then stained. Specifically, the grid was floated on a drop of the VLP solution for 3 min and dried with filter paper. After drying, the grid was floated on phosphotungstic acid staining solution for 30 s. The excess solution was then removed using filter paper. Finally, the dry grid was examined under an HT7700 microscope (Hitachi, Tokyo, Japan).

### Mice immunisation

Seven-week-old female *BALB/c* mice were purchased from Hangzhou Ziyuan Experimental Animal Technology Co. Ltd. They were housed in an SPF animal facility at Anhui Agricultural University, in accordance with the guidelines and regulations set forth by the Animal Care and Use Committee of Anhui Agricultural University (SYXK (Anhui) 2016-007). The mice were allowed one week to acclimatize to their new environment before the start of the experiment.

All experimental procedures adhered strictly to the guidelines of the Animal Care and Use Committee of Anhui Agricultural University. On days 0 and 28, a subcutaneous injection of 50 µg of AP205-S1 vaccine, S1 protein, or AP205-SpyCatcher was administered in the lateral abdominal area. Blood and fecal samples were collected weekly until day 49.

Tail vein blood was collected without anaesthesia, and at day 49, the mice were euthanised using a controlled CO_2_ atmosphere chamber. The sera were separated from the blood, and fecal samples were suspended in PBS at a ratio of 0.1 g per 1 mL of PBS. Both the sera and faecal supernatants were then collected and stored at −20 °C.

### IgG antibody measurement

The levels of IgG antibodies in serum and feces were determined using an ELISA (enzyme-linked immunosorbent assay). 96-well plates were coated with S1-SpyTag protein at 1 µg/mL in PBS overnight at 4 °C. Afterwards, the plates were washed four times with PBS. PBS-1% Casein was added to block plates for 2 h at room temperature. Following this, sera or fecal supernatants were added to the plates and serially diluted in PBS-1% Casein, allowing them to incubate for 1 h.

Next, the plates were washed four times with PBS-0.1% Tween. Goat anti-mouse IgG-HRP (Makewonderbio, Beijing, China) or goat anti-pig IgG-HRP (Cellwaylab, Luoyang, China) was incubated for 1 h to detect the bound IgG antibodies. Finally, TMB developing solution (Makewonderbio, Beijing, China) was added, followed by the same volume of 1 M H_2_SO_4_ solution to stop the reaction. The plates were read at OD_450 nm_ using a microplate spectrophotometer (Molecular Devices, California, USA). Antibody titres were calculated as the dilution folds that reached half of the OD_max_.

### IgA antibody measurement

IgG may compete with IgA antibodies for S1 binding sites; therefore, IgG should be removed when determining IgA responses. The serum or feces supernatant was first incubated with Protein A beads (Senhui Microsphere Tech, Jiangsu, China) for 30 min at room temperature, after which the supernatant was collected for IgA detection. Most procedures followed the same protocol for the IgG ELISA, except that the samples were pretreated, and the detection antibody used was goat anti-mouse IgA conjugated with HRP (Abcam in China, Shanghai, China). The binding curve was constructed using OD_450 nm_ and dilution folds.

### PEDV amplification and titre determination

The PEDV-KB2013 viruses were amplified in Vero cells grown in DMEM medium. When the cells reached 80% confluence on the plates, the virus was seeded and incubated for 1 h. Afterwards, the virus was removed by washing the cells with PBS three times. After culturing for 48 h, cytopathic changes were observed, including cell rounding, cluster formation, syncytium development and detachment from the plates. The infected cells were then collected after trypsinisation and stored at −80 °C.

Viral titres were determined using a plaque formation assay. A monolayer of Vero cells (100% confluent) was infected with thawed virus-containing supernatant, which had been serially diluted. The infection lasted for 1 h, after which the cells were incubated at 37 °C in a 5% CO_2_ atmosphere until visible plaques appeared. The titres were calculated, resulting in a viral titre of 5.75 × 10^5^ PFU/mL.

### Indirect immunofluorescence assay (IFA)

In addition to ELISA, an IFA was conducted to evaluate whether the immunised sera could recognise and bind to natural viruses. First, 200 TCID_50_ of the virus was added to 70% confluent Vero cells in a 24-well plate for 1 h. Twenty-four hours later, the cell supernatants were removed, and the cells were washed three times with PBS. Next, the cells were fixed with 4% paraformaldehyde for 30 min at room temperature and permeabilised with FoxP3/Transcription Factor Staining Buffer Kit (MultiSciences Biotech Co., Ltd, Hangzhou, Zhejiang, China).

Following this, a solution of PBS-1% Casein was applied to block additional binding sites on the plate for 2 h at 37 °C. Diluted sera were then added and incubated at 37 °C for 1 h. After washing the cells three times with PBS, a detection antibody, either goat-anti-mouse IgG-FITC (Biosharp, Anhui, China) or mouse-anti-pig IgG-FITC (CELLWAYLAB, Henan, China), was added and incubated with the cells at 37 °C for 1 h. Finally, the cell nuclei were stained with DAPI at room temperature for 10 min, and the images were captured under a fluorescent microscope.

### Neutralising assay

Cytopathic effects (CPE) caused by pretreated PEDV viruses were utilised to assess the viral neutralising capabilities of immunised sera. First, the sera were inactivated by heating at 56 °C for 30 min. Then the inactivated sera were incubated with PEDV for 1 h at 37 °C, using 1:2 serial dilutions starting from 1:20. The resulting reaction mixtures were added to Vero cells (70% confluent) and incubated for 1 h at 37 °C. After incubation, the mixtures were removed, and the cells were washed three times with PBS. The cells were then cultured for an additional 48 h, during which the CPE was observed under a microscope. The neutralising titres were recorded as the dilution folds necessary to completely neutralise viruses.

To visualise the neutralising effects of immunised sera, we performed an IFA. Instead of using the PEDV virus alone, we used immunised sera that had been pre-incubated with the virus to infect Vero cells. Additionally, we added polyclonal antibodies derived from rabbits against the PEDV-N protein (self-made) to identify infection-competent viruses. To detect these viruses, we used goat-anti-rabbit IgG-FITC, which indicates the quantity of infected viruses.

### ELISpot assay

To analyse the plasma cell counts in immunised mice, spleens were harvested on day 49 following the euthanasia of the mice. A single-cell suspension was obtained by crushing the spleens through a 70 µm strainer. Next, erythrocytes were removed by suspending the cells in ACK buffer for 5 min at room temperature. After this, an equal volume of medium was added, and the cells were centrifuged at 300 *g* for 5 min. The cells were then resuspended in RPMI 1640 medium supplemented with 10% FBS, and specific counts of cells were seeded into a 96-well Multiscreen plate (MilliporeSigma, Massachusetts, USA).

Before seeding the cells, the plate was coated with 10 µg/mL S1 protein and incubated overnight at 4 °C. The plate was then blocked using PBS-1% Casein at 37 °C for 2 h. The cells were discarded after a 5 h incubation at 37 °C in a 5% CO_2_ atmosphere. The primary antibody, Goat Anti-Mouse IgG (ThermoFisher, Massachusetts, USA), was then incubated overnight at 4 °C. Next, the secondary antibody, Donkey Anti-Goat IgG-AP (Biosharp, Anhui, China), was incubated at room temperature for 2 h. Finally, the spots were visualised using the BCIP/NBT substrate kit (Biosharp, Anhui, China).

### Immunisation of piglets and viral challenge

The neonatal piglets used in this study were housed at Northwest Agriculture & Forestry University's experimental animal centre. The Animal Welfare Committee of Northwest A&F University reviewed and approved the experiment protocol. Throughout the study, all animals were monitored daily for clinical signs.

At seven days of age, the piglets were primed (day -21) and boosted (day -7) with 100 µg of either AP205-S1 or AP205-SpyCatcher, administered via intramuscular injection in the neck area using 200 µL MONTANIDE ISA 206 VG (Seppic Shanghai Chemical Specialities, Shanghai, China). On day 0, the animals were orally challenged with 1 × 10^5^ TCID_50_ KB2013. Sera and rectal swabs were collected 0, 3, 5, 7, 10 and 14 days after the challenge. Each group consisted of 4 randomly assigned individuals from the same litter.

The severity of diarrhoea was assessed as follows: normal and soft feces were scored as 0, loose faeces as 1, and watery faeces as 2. All animals were euthanised on day 14 post-challenge through a bleeding out procedure from the axillary artery of the forelimb, performed under deep anasthesia using an intramuscular injection of 5 mg/kg xylazine. It is important to note that one animal in the AP205-S1 group died from congenital hypoplasia at day 4.

### RT-qPCR

The loads of PEDV in the serum and feces of challenged piglets were assessed using RT-qPCR. Following the manufacturer's instructions, the total RNA was isolated from rectal swabs using the Viral Genome Extraction Kit (Qingdao Lijian Bio-Tech, Qingdao, China). Next, cDNA was synthesised from the RNA using the HyperScript III 1st Strand cDNA Synthesis Kit with gDNA Remover (EnzyArtisan Biotech, Shanghai, China), which served as a template for the qPCR.

The reaction system was prepared as follows (20 µL): 10 µL SYBR Green, 8 µL ddH_2_O, 0.5 µL Primer-F (10 µM), 0.5 µL Primer-R (10 µM), 1 µL cDNA.

The primer sequences used were F: GAGGGTGTTTTCTGGGTTG, R: CGTGAAGTAGGAGGTGTGTTAG.

To establish a standard curve, the target gene (N gene) was constructed on pET28a and the Ct = −3.225Log_10_ (Copies) + 36.87.

### Statistical analysis

The significance analysis was conducted using GraphPad Prism 9 (GraphPad Software, Inc., La Jolla, CA, USA). The *P* values from the unpaired t-test were categorised as follows: ≤ 0.05 (*), ≤ 0.01 (**), ≤ 0.001 (***), ≤ 0.0001 (****). All error bars were displayed as mean ± SEM.

## Results

### SpyCatcher/Tag bond effectively conjugates AP205-VLP and the S1 protein without compromising the advantages of VLP

**Figure 1:**
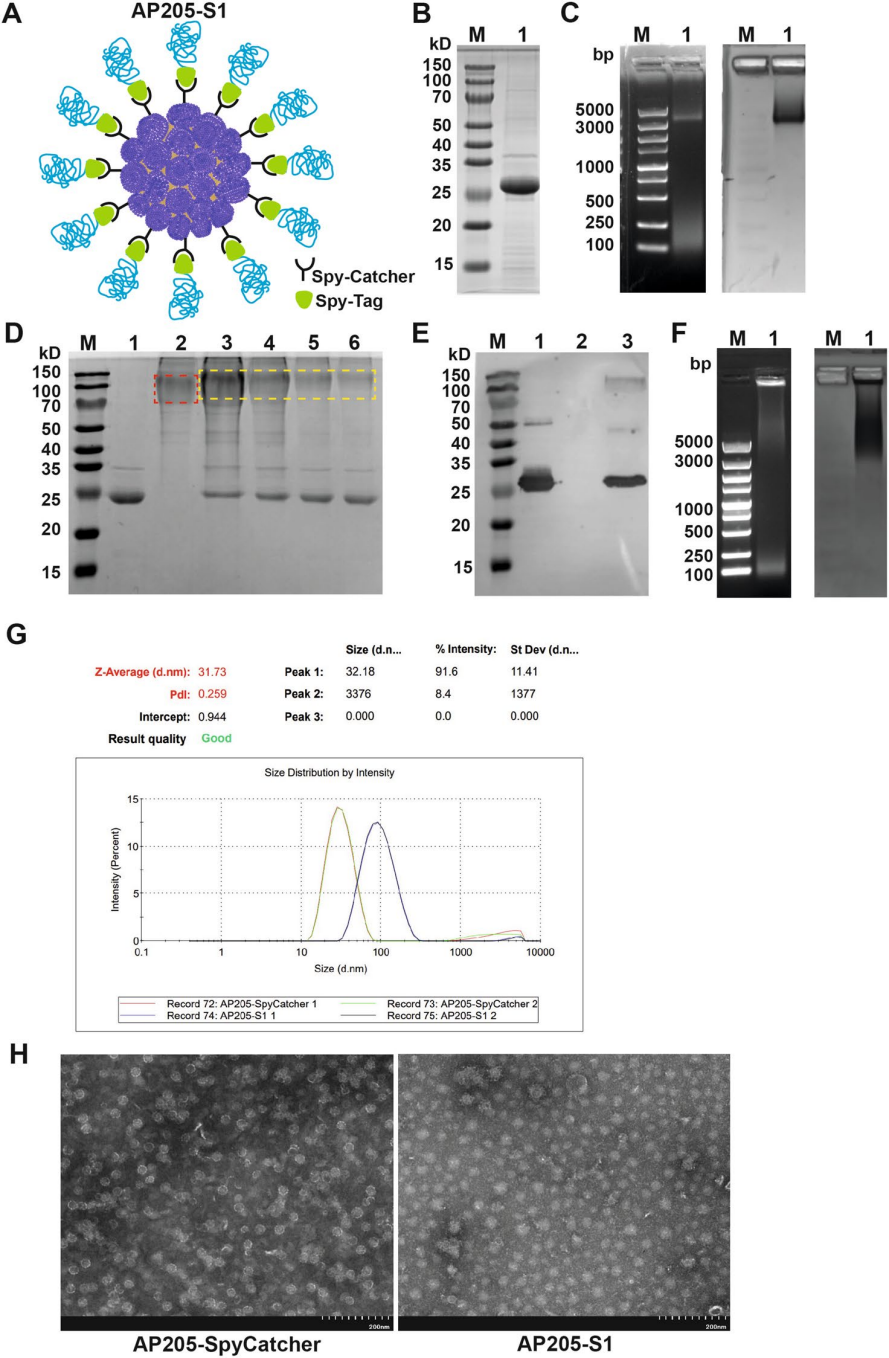
**Generation and characterisation of the AP205-S1 vaccine.** **A** Diagram of the way displaying S1 protein on surface of AP205-VLP via SpyTag and SpyCatcher specific bonds; **B** SDS-PAGE image of purified AP205-SpyCatcher (lane 1); **C** Agarose gel images of purified AP205-SpyCatcher (lane 1), left: UV light, right: Coomassie blue staining; **D** SDS-PAGE image of different molar ratios of AP205-SpyCatcher to S1-SpyTag, lane 1: AP205-SpyCatcher alone, lane 2: S1-SpyTag alone; lane 3: 1:2, lane 4: 1:1, lane 5: 2:1, lane 6: 3:1; **E** Western blot image of the generated AP205-S1 using monoclonal anti-AP205 antibody, lane 1: AP205-SpyCatcher alone, lane 2: S1-SpyTag alone; lane 3: AP205-S1 (1:1 reaction); **F** Agarose gel images of the formed AP205-S1 (lane 1), left: UV light, right: Coomassie blue staining; **G** Dynamic light scattering reports of AP205-SpyCatcher and formed AP205-S1; **H** TEM images of AP205-SpyCatcher and AP205-S1.

Chemical coupling was the first method attempted to link the purified AP205-VLP with the S1 protein. However, this approach was inefficient, likely due to the relatively large molecular size of the S1 protein (data not shown). Consequently, we sought to produce an AP205-S1 vaccine using specific binding via SpyCatcher/Tag, as illustrated in Figure 1A.

Initially, AP205-SpyCatcher was successfully expressed and purified, exhibiting a molecular weight of 26 kD per subunit, as shown in Figure 1B. Furthermore, agarose gel analysis confirmed that ssRNA was packaged within AP205-SpyCatcher (Figure 1C). Although the molecular size of SpyCatcher (12 kD) is comparable to that of the AP205 monomer (14 kD), the AP205-SpyCatcher effectively retained the viral particle (Figure 1H).

After purifying the S1-SpyTag expressed by HEK293 cells, we generated AP205-S1 by incubating AP205-SpyCatcher with S1-SpyTag. We tested different molecular ratios between AP205-SpyCatcher and S1-SpyTag to determine the optimal reaction conditions. In the SDS-PAGE gel, the band corresponding to AP205-S1 was slightly lifted above the S1-SpyTag at all tested ratios (Figure 1D). Additionally, the band corresponding to AP205-SpyCatcher faded when mixed with S1-SpyTag. We observed the highest coupling efficiency of 49% at a ratio of 1:1, which allowed us to generate the AP205-S1 vaccine for further characterisation and immunisation.

Western blot results using the AP205-specific monoclonal antibody confirmed the formation of a covalent bond, as shown in Figure 1E. Instead of presenting a distant RNA band for AP205-SpyCatcher, the RNA in AP205-S1 appeared diffuse but co-localised with the protein band after Coomassie blue staining (Figure 1F). Additionally, DLS demonstrated that the AP205-S1 particles were homogenous, with a diameter of 80 nm (Figure 1G). TEM revealed that the vaccine exhibited a viral particle structure (Figure 1H).

These results suggested that AP205-S1, connected by a SpyCatcher/Tag bond, was homogeneous, maintained a stable spherical shape, and was packaged with prokaryotic ssRNA.

### AP205-S1 vaccine exhibits robust immunogenicity in mice

**Figure 2:**
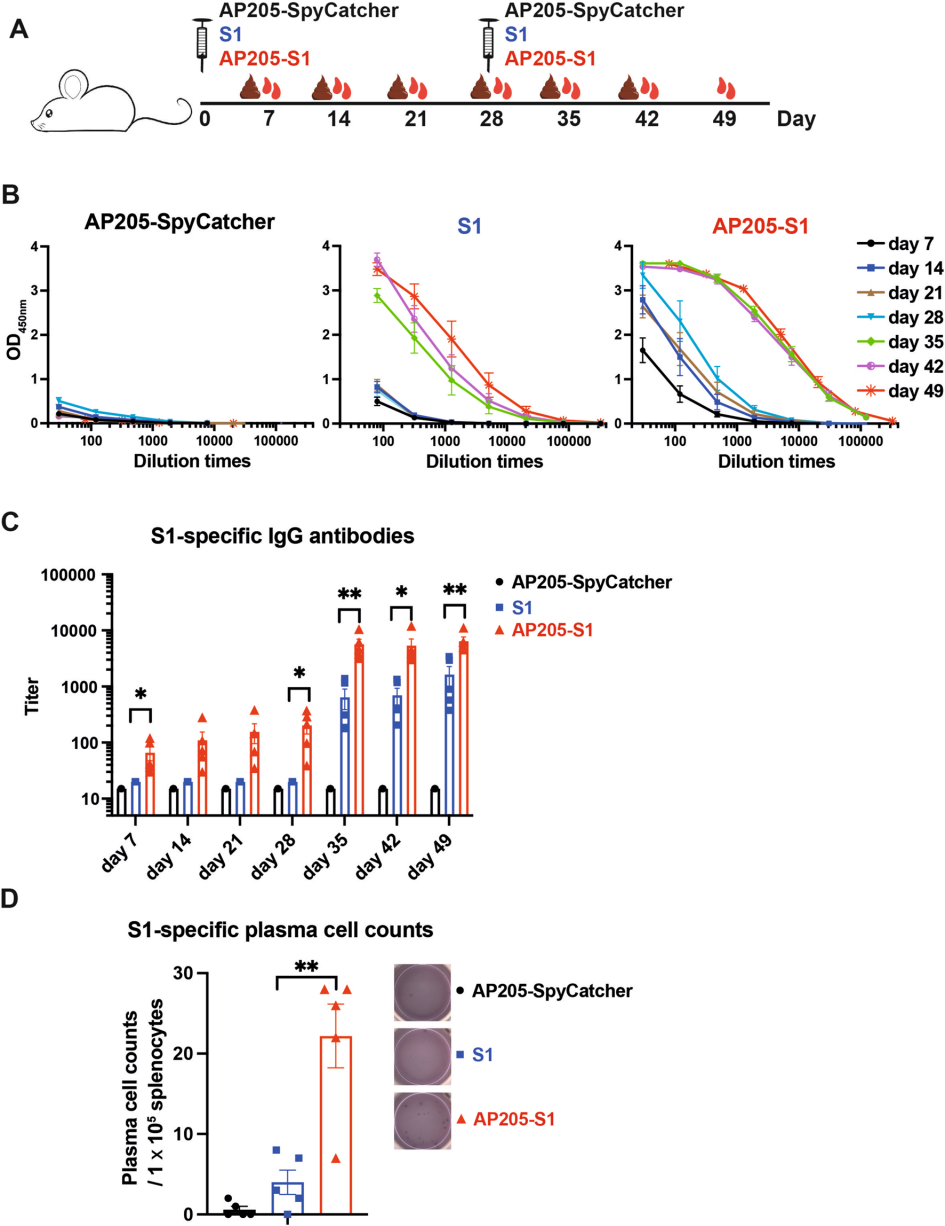
**S1-specific IgG antibody responses in mouse sera after immunisation.** **A** Mice immunization regimen. Serum and feces samples were collected weekly. **B** ELISA results of AP205-SpyCatcher, S1 or AP205-S1 immunised mice sera against S1 protein at different time points. Each point represented 5 mice, and the error bar was shown as ± SEM. **C** S1-specific IgG antibody titres of immunised sera at different time points. Each point represented one mouse, and the error bar was shown as ± SEM. **D** Plasma cell counts that secrete S1-specific IgG antibodies in immunised spleens at day 49. One well of spots was displayed in each group. *P* ≤ 0.05 was shown *, *P* ≤ 0.01 as **.

To examine the ability of AP205-S1 to induce immune responses in vivo, *BALB/c* mice were primed on day 0 and boosted on day 28 (Figure 2A). The levels of IgG antibodies in the sera were assessed using ELISA, which showed that mice immunised with AP205-S1 produced S1-specific IgG antibodies as early as day 7 (Figure 2B). The IgG levels increased over time, with a notable rise after the booster shot, peaking on day 35 and remaining elevated until day 49. In contrast, mice immunised with the S1 protein only demonstrated significant antibody levels following the second injection. As anticipated, AP205-SpyCatcher did not induce S1-specific antibody responses even after two injections. The S1-specific IgG titres elicited by AP205-S1 were significantly higher than those caused by S1 or AP205-SpyCatcher alone, indicating that the display of S1 on AP205 greatly enhanced its immunogenicity (Figure 2C). Furthermore, AP205-S1 stimulated significantly more plasma cells secreting S1-specific IgG antibodies than S1 and AP205-SpyCatcher on day 49, as shown by the ELISpot assay (Figure 2D).

**Figure 3:**
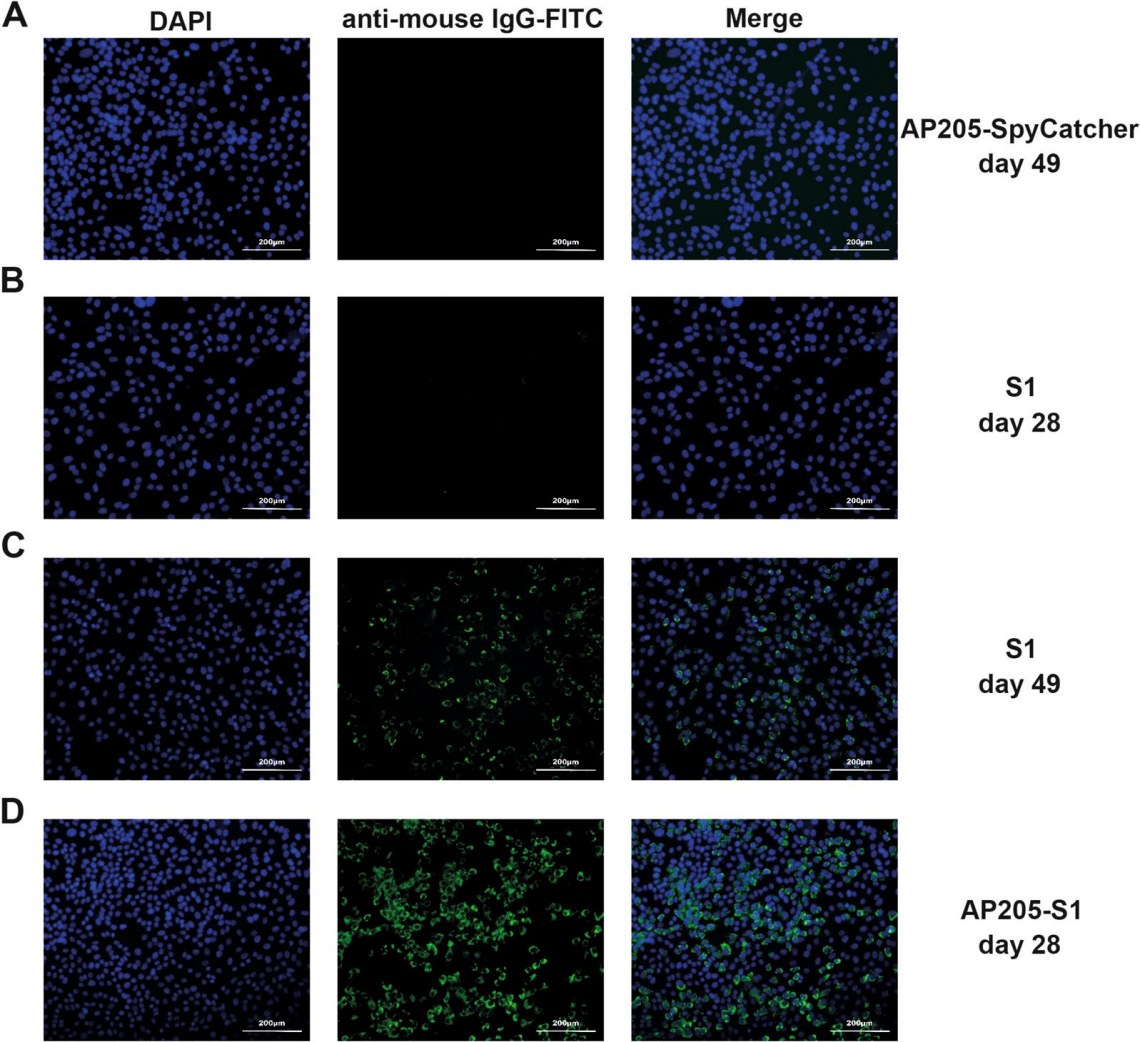
**IFA images of immunised mice sera to recognise PEDV KB2013 viruses in vitro.** **A** Day 49, mouse serum immunised with AP205-SpyCatcher. **B**, **C** mice sera immunised with S1 protein at days 28 (**B**) and 49 (**C**). D: Mouse serum immunised with AP205-S1 (day 28). DAPI-stained nucleus and FITC reflected mouse IgG antibodies bound to viruses. Serum samples at day 49 and day 28 were diluted 1:1000 or 1:100, respectively.

Additionally, an IFA was conducted to assess whether the immunised sera could recognise and bind to PEDV-infected cells in vitro. In line with the ELISA results, sera from the AP205-SpyCatcher immunisation at day 49 showed no binding to PEDV-infected cells, as indicated by the absence of FITC signals (Figure 3A). For the S1 immunisation, the sera collected on day 28 did not bind to PEDV, while the sera from day 49 demonstrated binding, reflecting the increased antibody titres following the booster (Figures 3B and C). Moreover, the IgG antibodies elicited by the AP205-S1 immunisation on day 28 showed the ability to bind to PEDV viruses in vitro, as illustrated in Figure 3D. These findings suggest that the S1 protein displayed on AP205-VLPs maintained its natural conformation and indicate that AP205-S1 effectively stimulated robust immune responses in mice.

### Mice immunised with AP205-S1 produce high-affinity S1-specific IgG antibodies

**Figure 4:**
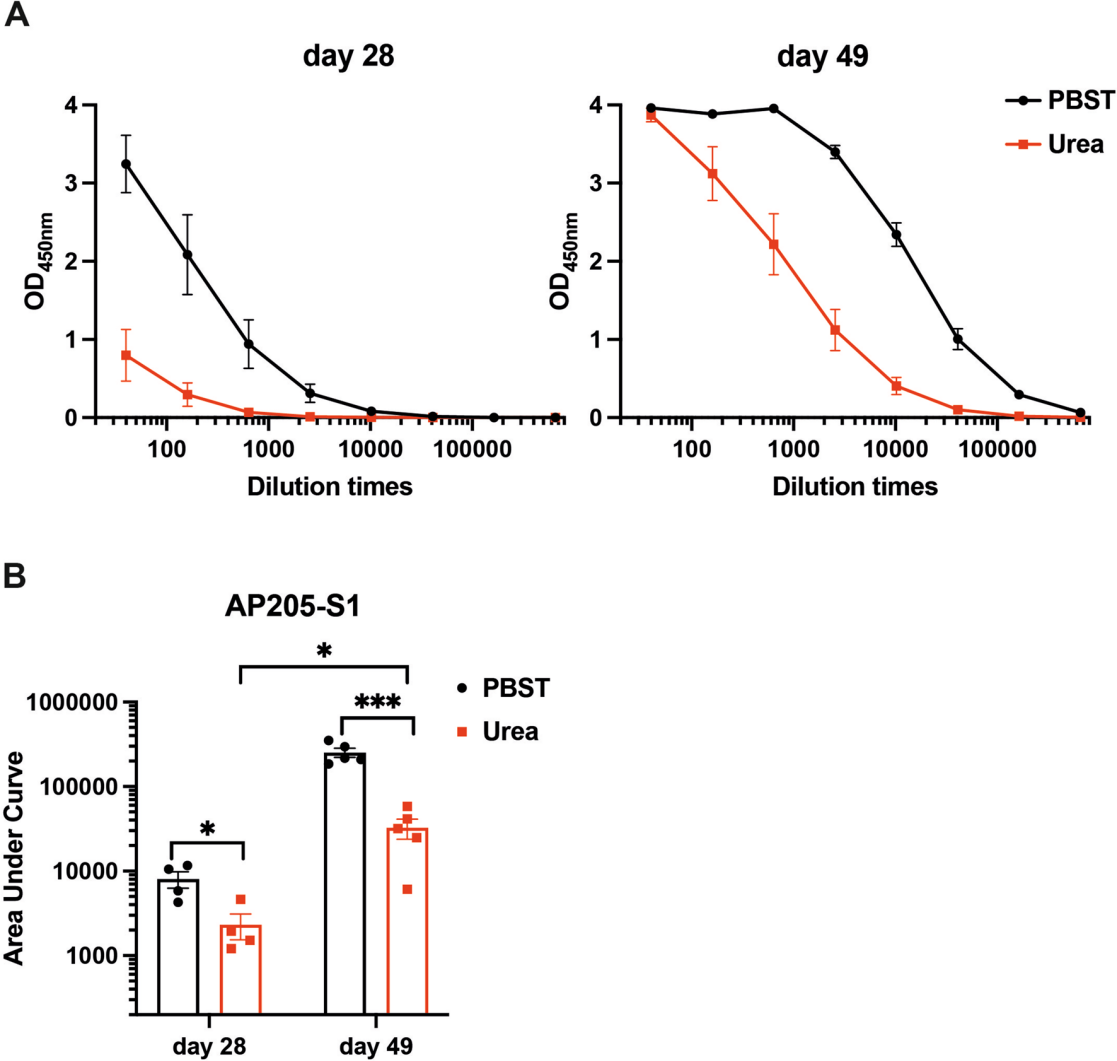
**Avidity ELISA results of immunised mice sera against S1 protein**. **A** ELISA curves of OD_450nm_ values with diluted sera washed with(red) or without (black) 7 M urea. **B** Area under the curve values of avidity ELISA curves. *P* ≤ 0.05 was shown *, *P* ≤ 0.001 as ***.

High binding strengths between antibodies and viruses are correlated with their neutralising potentials. An avidity ELISA was performed to assess the levels of high-avidity antibodies. The principle of avidity ELISA is that those antibodies bound weakly to the S1 protein are washed away using 7 M urea, while high-avidity antibodies remain attached to the plates. As anticipated, the quantity of high-avidity antibodies was significantly lower than the total IgG levels, as shown in Figure 4A. Consistent with the total IgG levels, high-avidity IgG antibodies were relatively low before the booster dose (day 28). In contrast, the levels of high-avidity IgG antibodies significantly increased after the booster dose on day 49 (Figure 4B), indicating that affinity maturation improved following the booster. These results suggest that AP205-S1 can induce high-avidity antibodies that may effectively neutralise PEDV.

### AP205-S1 induces systemic IgA antibodies targeting the S1 protein

**Figure 5:**
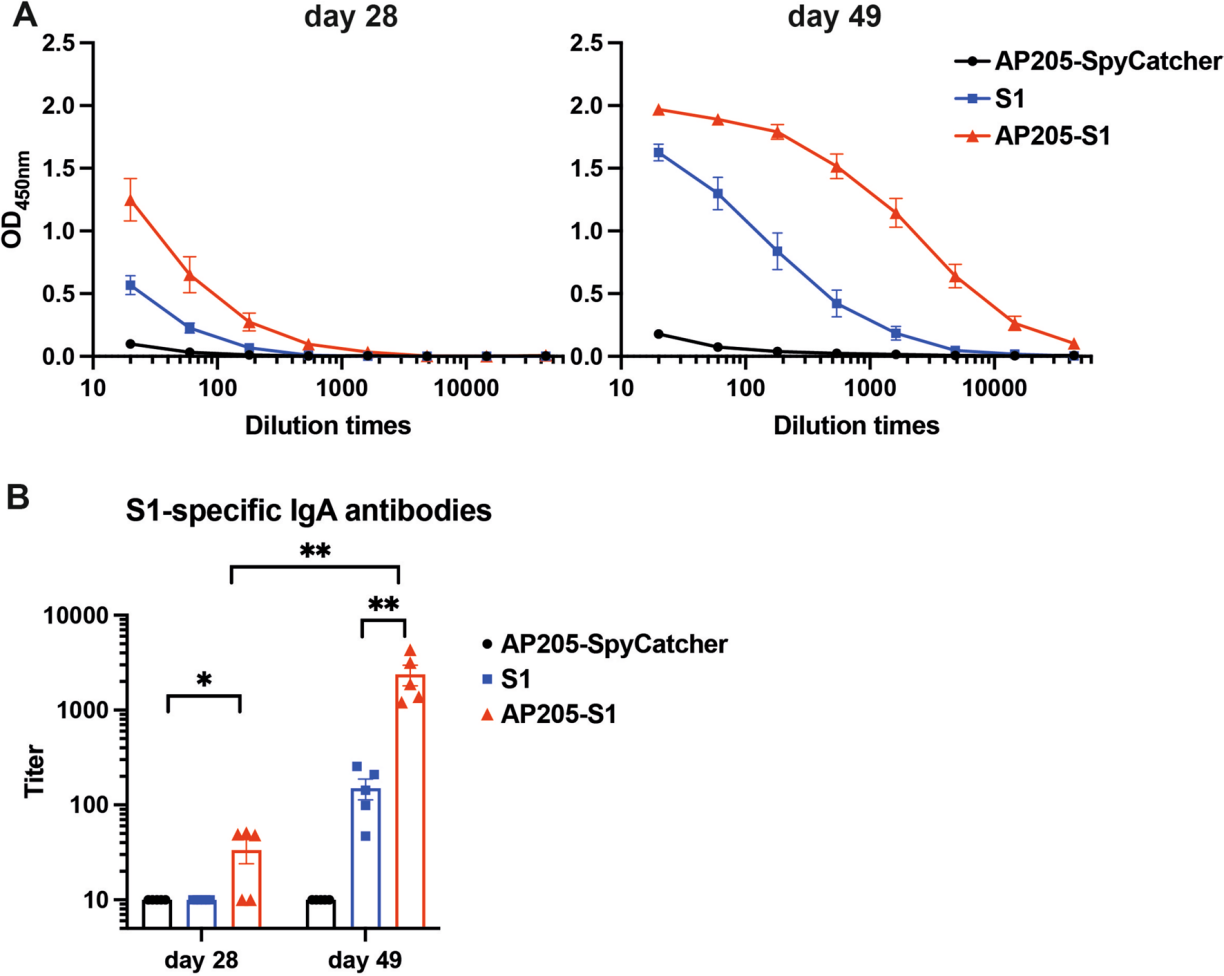
**S1-specific IgA antibody levels in immunised mice sera**. **A** ELISA curves of day 28 and 49 sera. Each point represented 5 mice, and the error bar was shown as ± SEM. **B** S1-specific IgA titres of each group at days 28 and 49. *P* ≤ 0.05 was shown *, *P* ≤ 0.01 as **.

IgA antibodies play a crucial role in controlling viruses, particularly in the mucosa, alongside IgG antibodies. Following the initial immunisation, AP205-S1 and S1 stimulated S1-specific IgA antibodies (Figure 5A). Similar to the IgG levels, the levels of IgA antibodies in sera were increased after the booster shot, with AP205-S1 showing greater immunogenicity than the S1 protein regarding IgA antibody production (Figure 5B). However, AP205-SpyCatcher did not induce S1-specific IgA antibodies in the mice's sera, even after two immunisations.

While IgA levels in the sera can reflect those in mucosa to some extent, IgA antibodies were not detected in the fecal supernatants of any immunised mice (data not shown). This indicates that systemic immunisation did not successfully induce mucosal IgA antibodies. Nonetheless, systemic IgA antibodies, especially the S1-specific IgA, have demonstrated excellent viral neutralising effects in PEDV-infected pigs.

In conclusion, AP205-S1 effectively induced high levels of systemic IgA antibodies in mice upon systemic administration, which is likely to neutralise PEDV.

### Sera from mice immunised with AP205-S1 exhibit strong neutralising capabilities against PEDV

**Figure 6:**
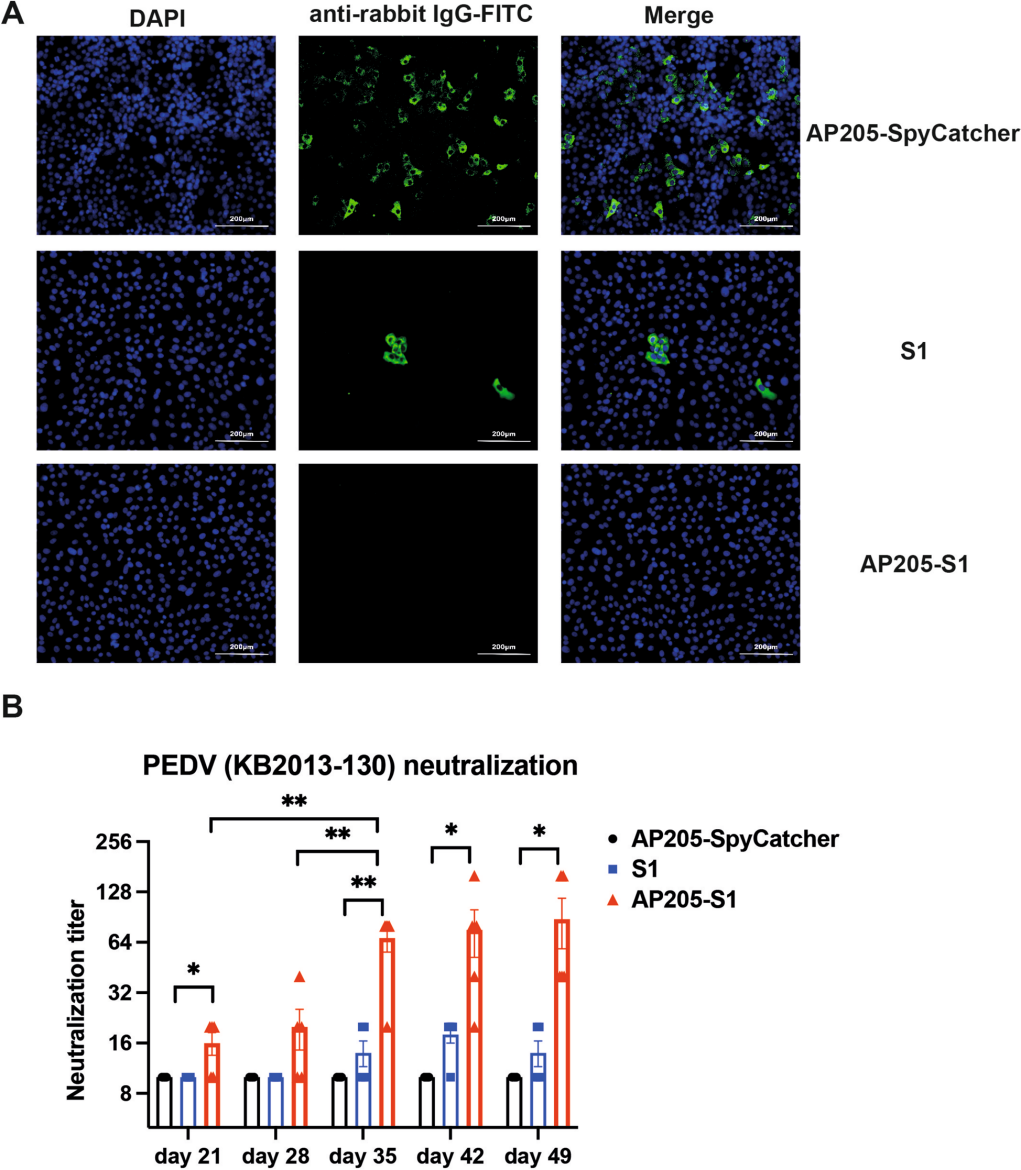
**Immunised mice sera to neutralise PEDV (KB2013) viruses in vitro**. **A** IFA images of day 49 sera immunised with AP205-SpyCatcher, S1 protein or AP205-S1 to block viruses from infecting Vero cells. DAPI-stained nucleus and FITC reflected viral N protein-specific antibodies derived from rabbit, i.e. the infected viruses. AP205-SpyCatcher immunised sera were diluted 1:10, and S1 protein and AP205-S1 immunised sera were diluted 1:100. **B** Neutralisation titres of immunised sera from day 21 to day 49. The dilution folds of sera to completely (100%) inhibit CPE in Vero cells were determined as titres. *P* ≤ 0.05 was shown *, *P* ≤ 0.01 as **.

Next, the abilities of AP205-S1 to elicit antibodies that neutralise PEDV viruses were assessed. The viruses were first incubated with immunised sera and added to Vero cells. IFA results demonstrated that AP205-SpyCatcher immunised sera failed to neutralise the KB2013 virus. Thus, FITC signals were detected as shown in Figure 6A. In contrast, AP205-S1 immunised mice sera completely neutralised the PEDV virus from infecting Vero cells (Figure 6A). Notably, S1 elicited antibodies partly neutralised PEDV, indicating that the S1 protein could induce neutralising antibodies but with strongly delayed kinetics and lower levels.

CPE assays were conducted to assess the neutralising titers of sera from vaccinated individuals. As expected, sera from AP205-SpyCatcher immunised subjects exhibited no neutralising effects. In contrast, sera from S1 immunised subjects demonstrated a slight viral neutralising capacity after the booster dose. Notably, sera from AP205-S1 immunised subjects showed viral neutralising effects at all tested time points. Consistent with the antibody titres, the neutralisation titres of sera from AP205-S1 immunised individuals were significantly elevated after the booster, with some samples reaching 1:160 by day 49 (Figure 6B). In summary, both IFA and CPE assays confirmed that AP205-S1 induced the production of neutralising antibodies.

### AP205-S1 stimulates neutralising mucosal IgG antibodies in mice

**Figure 7:**
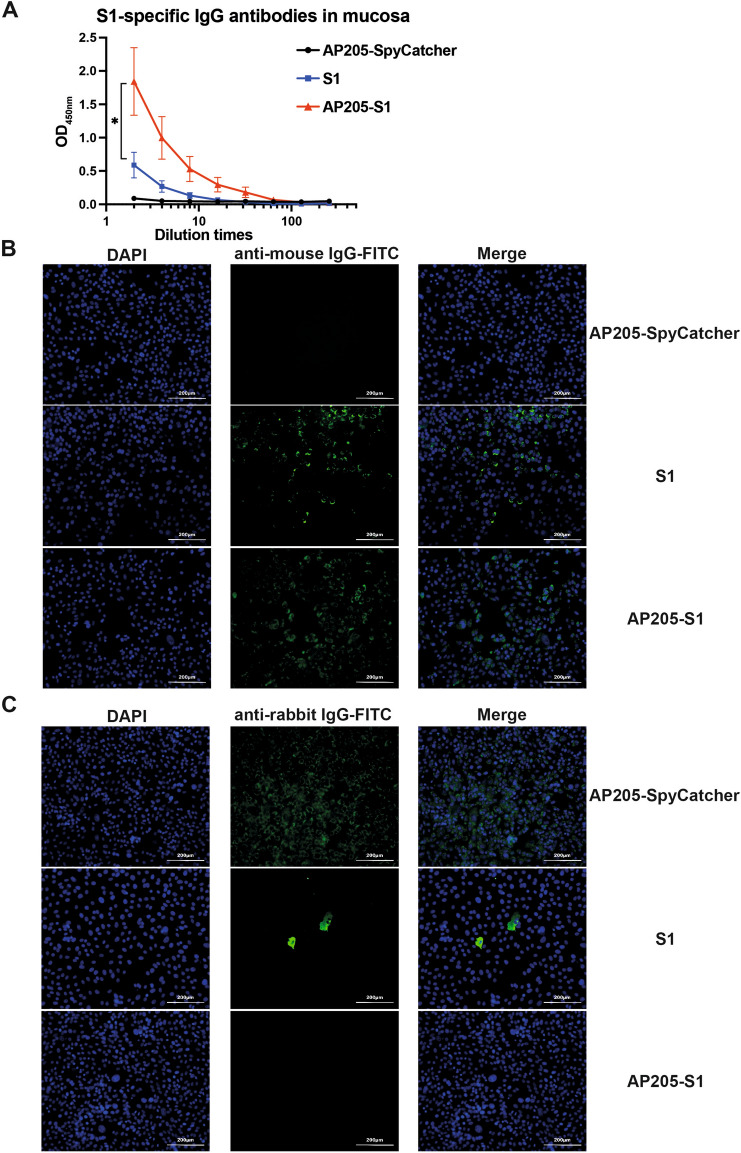
**Antibody responses in the intestinal mucosa of mice at day 42 after prime immunisation**. **A** ELISA curves of S1-specific IgG antibodies in faecal supernatants. **B** IFA images of mouse faecal supernatants to bind to PEDV (KB2013) viruses in vitro. DAPI-stained nucleus and FITC reflected mouse IgG antibodies bound to viruses. **C** IFA images of mouse faecal supernatants to neutralise PEDV (KB2013) viruses from infecting Vero cells. DAPI-stained nucleus and FITC reflected viral N protein-specific antibodies derived from rabbit, i.e. the infected viruses. Faecal supernatants were not diluted. *P* ≤ 0.05 was shown *.

Since PEDV is an enteric virus, local gastrointestinal antibodies initially play a crucial role in defending against viral infections. To determine whether mucosal antibodies were generated following immunisation, faecal supernatants from mice were analysed using ELISA. The results indicated that mucosal IgG antibodies were induced by both AP205-S1 and the S1 protein following a booster dose (Figure 7A). Notably, AP205-S1 induced significantly higher mucosal S1-specific IgG antibody levels than the S1 protein. Overall, the mucosal IgG levels in mice immunised with either AP205-S1 or S1 were considerably lower than in serum. However, the mucosal antibodies did specifically bind to natural PEDV viruses in vitro, as shown in Figure 7B. Furthermore, mucosal antibodies induced by AP205-S1 completely blocked PEDV from infecting Vero cells, while mucosal antibodies from the S1 protein only partially inhibited the infection (Figure 7C). These results indicate that the systemic administration of AP205-S1 effectively induces superior systemic and mucosal antibody responses, both of which neutralise PEDV efficiently.

### Systemic and mucosal antibodies induced by AP205-S1 show cross-reactivity with AH2018-HF1 (G-I strain)

One significant challenge in controlling PEDV is the immune escape of emerging variants. This challenge could potentially be addressed by developing vaccines that provide broad cross-protection. To determine whether antibodies induced by AP205-S1 could recognise and neutralise other PEDV strains, we tested the AH2018-HF1 strain (G-I strain), which has an S1 protein with 88.5% similarity to that of PEDV-KB2013 (Additional file 1).

**Figure 8:**
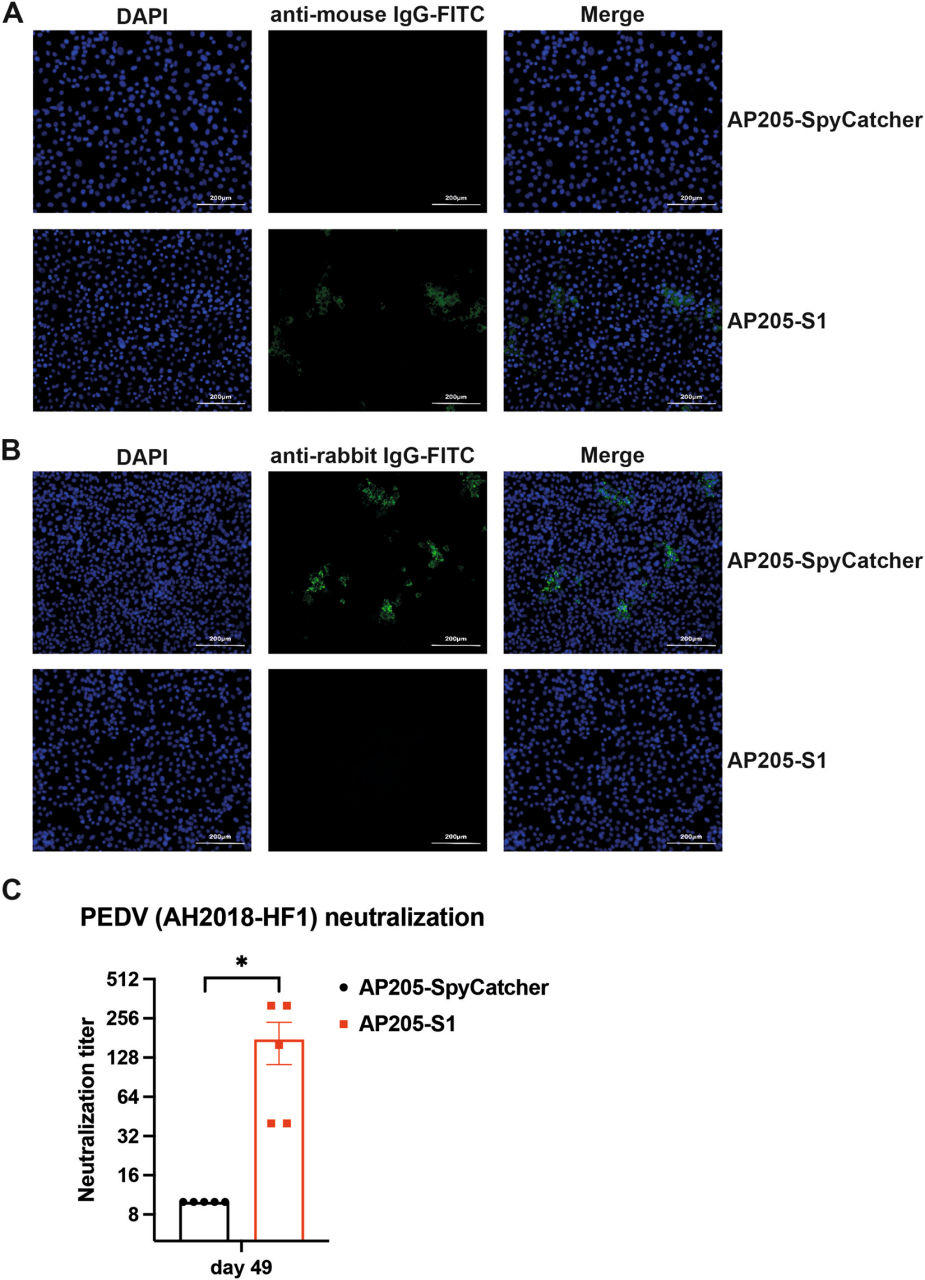
**Recognition and neutralisation of immunised mice sera to PEDV AH2018-HF1 (G-I strain) virus**. **A** IFA images of mouse sera at day 49 to bind to AH2018-HF1 viruses in vitro. DAPI-stained nucleus and FITC reflected mouse IgG antibodies bound to viruses. Serum samples were diluted 1:1000. **B** IFA images of mouse sera at day 49 to neutralise AH2018-HF1 viruses from infecting Vero cells. DAPI-stained nucleus and FITC reflected viral N protein-specific antibodies derived from rabbit, i.e. the infected viruses. Serum samples were diluted 1:10. **C** Neutralisation titres of immunised sera at day 49. The dilution folds of sera to completely (100%) inhibit CPE in cells caused by AH2018-HF1 infection were determined as titres. *P* ≤ 0.05 was shown *.

Results from the IFA indicated that serum samples from mice immunised with AP205-S1 (day 49) were able to bind to AH2018-HF1 in vitro (Figure 8A), which could neutralise the virus as well (Figure 8B). Furthermore, the neutralising titres against AH2018-HF1 in the sera of immunised mice were significantly higher than those immunised with AP205-SpyCatcher (Figure 8C). This suggests that AP205-S1 can induce cross-neutralising antibodies against a G-I strain of the virus.

**Figure 9:**
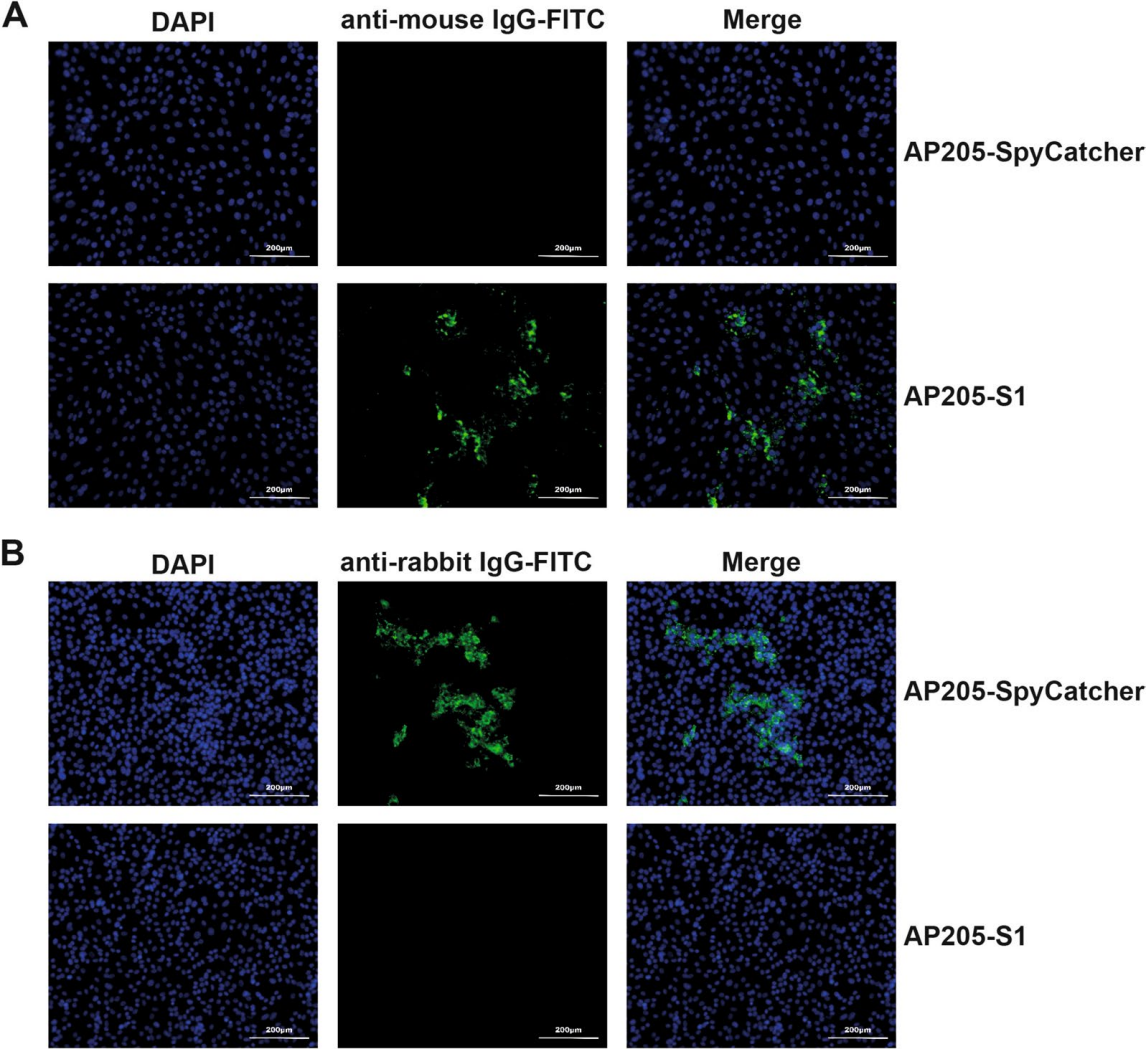
**Mucosal antibodies from immunised mice to recognise and neutralise the PEDV AH2018-HF1 (G-I strain) virus**. **A** IFA images of mouse faecal supernatants on day 42 to bind to AH2018-HF1 viruses in vitro. DAPI-stained nucleus and FITC reflected mouse IgG antibodies bound to viruses. **B** IFA images of mouse faecal supernatants on day 42 to neutralise AH2018-HF1 viruses from infecting Vero cells. DAPI-stained nucleus and FITC reflected viral N protein-specific antibodies derived from rabbit, i.e. the infected viruses. Faecal supernatants were not diluted.

Furthermore, we assessed whether the mucosal antibodies elicited by the AP205-S1 vaccine could cross-react with the AH2018-HF1 virus. The IFA results indicated that these antibodies could both recognise (Figure 9A) and neutralise (Figure 9B) the AH2018-HF1 virus. In conclusion, the vaccine derived from KB2013, AP205-S1, induced strong systemic and local antibody responses that neutralised the KB2013 virus and exhibited cross-reactivity with the G-I strain, AH2018-HF1.

### Immunising piglets with AP205-S1 protects against PEDV infection

**Figure 10:**
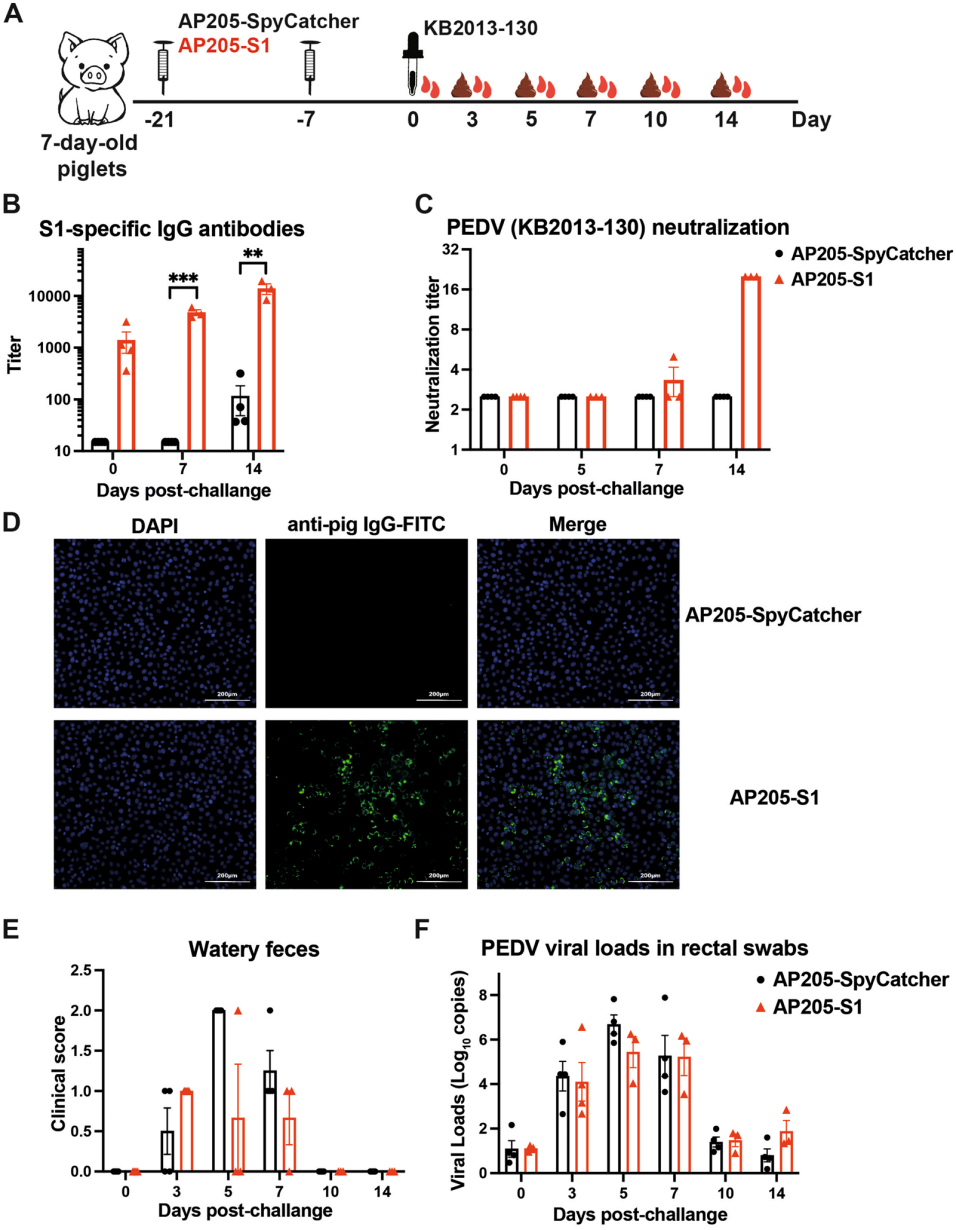
**Protection efficacy of AP205-S1 in piglets against PEDV infection after AP205-S1 immunisation.** **A** Immunisation and challenge regimen of pig experiments. **B** S1-specific IgG titres in pig sera after challenge. **C** Neutralisation titres of pig sera after challenge. **D** IFA results of day 0 pig sera to bind to PEDV in vitro. Sera were diluted 1:1000. DAPI-stained nuclei and FITC reflected pig IgG antibodies bound to viruses. **E** Diarrhoea scores of piglets after challenge. **F** PEDV viral loads in pig rectal swabs after challenge.

The results clearly demonstrated that AP205-S1 elicited neutralising antibodies in both serum and mucosal samples of mice, and we aimed to evaluate the protective effects in piglets following a PEDV challenge. Suckling piglets were immunised with AP205-S1 twice before being exposed to a lethal dose of the KB2013 virus when they were seven days old (Figure 10A). Following the booster immunisation, high levels of S1-specific IgG antibodies were detected in the serum (Figure 10B), which could recognise PEDV in vitro (Figure 10D). Additionally, immunised sera completely neutralised PEDV viruses, as shown in Figure 10C, indicating that AP205-S1 demonstrated excellent piglet immunogenicity.

Since the piglets were 28 days old at the time of the challenge, all animals survived, including those immunised with AP205-SpyCatcher (control). Overall, the piglets immunised with AP205-S1 exhibited more active movements (data not shown). Furthermore, from 5 to 7 days post-challenge, fewer instances of watery faeces were observed in the AP205-S1 immunised piglets, although the difference was not statistically significant (Figure 10E). Additionally, the viral loads in the immunised pigs were lower than those in the control pigs, but this difference was not significant (Figure 10F). This observation parallels the findings in COVID-19 vaccines, where a limited impact on viral spread was noted despite a marked reduction in disease severity.

In conclusion, AP205-S1 demonstrated modest prophylactic effects in piglets against PEDV infection; however, further development is needed for industrial application.

## Discussion

PEDV has been a significant pathogen in the pork industry for decades, and vaccines are currently under intense development worldwide. To address the emergence of new variants, vaccine candidates must be updated regularly, especially since 2010, when G-II strains became a significant concern. Typically, the S1 protein of coronaviruses binds specifically to receptors, while the S2 protein mediates membrane fusion, facilitating virus internalisation. Studies have shown that blocking the S1 and S2 activities can prevent virus invasion. For instance, the cocktail antibodies REGN-COV, which target the receptor binding domain (RBD) in the S1 region of SARS-CoV-2, have been effective in neutralising and reducing viral loads in patients [25, 26].

Additionally, serum antibodies from convalescent patients have been found to recognise an epitope located at the fusion peptide site of the S2 protein, which could potentially serve as a neutralising epitope [27]. Although porcine aminopeptidase N (pAN), sialic acid, C-type lectin, and heparan sulfate have been implicated in viral entry across different models, the host receptors for PEDV are not definitively defined. This uncertainty necessitates a focus on the coordination of protein–protein binding and glycan–protein binding [28, 29].

Following the unveiling of the PEDV S protein structure through Cyro-EM, the NTD and CTD within the S1 protein were identified as functional domains responsible for glycan and protein binding, respectively [17]. Therefore, it is evident that the S1 protein of PEDV plays a crucial role in binding to the cell receptor, making it an ideal target for vaccine development. Based on these insights, we have chosen the S1 protein as the target antigen for designing the PEDV vaccine.

Free proteins often lead to insufficient antigen presentation, which results in limited immune responses. Displaying targeting proteins on a VLP scaffold can enhance antigen-presenting signals due to several advantages: (i) The nanometer size of VLPs allows them to drain freely in lymphatic vessels, making them accessible to present antigens; (ii) The repetitive organisation of antigens on VLPs amplifies antigen-presenting signalling; (iii) The spacing between adjacent antigens on VLPs is ideal for BCR crosslinking; (iv) The nucleic acids packaged within VLPs stimulate innate immune responses, thereby facilitating specific adaptive responses [30–32].

In this study, the AP205-S1 vaccine exhibited a particulate shape with a diameter of approximately 80 nm and contained prokaryotic ssRNA, which was packed. Unlike the focused RNA band packed in AP205-SpyCatcher, the RNA packed in AP205-S1 was diffused, likely due to the disparate numbers of S1-SpyTag protein on each particle. Consequently, the observation that the AP205-S1 vaccine elicits strong antibody responses aligns with previous studies indicating that TLR signalling triggered by VLP-packaged ssRNA is crucial for antibody affinity maturation and the generation of secondary plasma cells [33, 34].

The structural proteins of PEDV, specifically the envelope (E) protein, membrane (M) protein, and spike (S) protein, have been shown to form VLPs when expressed externally in both baculovirus and mammalian cells. Immunising mice with these PEDV-VLPs, which are comprised of the E, M, and S proteins, can induce the production of IgG and IgA antibodies, as well as T cell responses [35]. When pigs were vaccinated with PEDV-VLPs, they developed both humoral and cellular immune responses, which provided some degree of protection against viral infection [36]. Additionally, a putative B cell epitope in the S protein, displayed on the Hepatitis B Core Antigen (HBcAg) VLP, stimulated the production of neutralising antibodies in mice and gilts [37].

The significant disadvantage of AP205-S1 is that the S1 protein was produced in eukaryotic cells, which can be relatively expensive. This may make large-scale production and veterinary applications challenging. However, we demonstrate that the S1 protein contains neutralising epitopes that can induce a neutralising antibody response against PEDV. To create more cost-effective PEDV vaccine candidates, we will explore alternative expression systems, such as yeast or insect cells, for producing the S1 protein. Alternatively, we may use a truncated version (RBD), which can be expressed in *E. coli*.

Furthermore, the costs associated with the large-scale production of proteins expressed in mammalian cells can be significantly reduced by optimising the expression system or by creating a stable-expression cell line. In fact, Guo et al. utilized an efficient expression system in HEK293F cells to produce the S protein, which exhibited excellent immunogenicity, eliciting strong humoral and cellular immune responses [38]. Additionally, Song et al. vaccinated pigs with bivalent subunit vaccines produced in eukaryotic cells, which successfully protected against both G-IIa and G-IIb strains [39].

Moreover, due to its relatively high efficiency, the SpyCatcher/Tag system will continue to be employed to link AP205-VLP and antigens. This reaction can be easily achieved by simply mixing and incubating the components under moderate conditions.

The mechanisms by which VLPs promote the generation of IgA have been reported. In a study using a humanised mouse model, Norovirus VLPs were found to trigger IgA responses that showed superior virus-neutralising activities [40]. In mice immunised with VLPs, IgA antibodies were successfully induced in serum through both systemic (subcutaneous) and mucosal (intranasal) administration [41].

These systemic and mucosal IgA antibody responses were shown to depend on TLR7 signalling in B cells, as well as in lung DCs and alveolar macrophages [42, 43]. Additionally, AP205-S1 strongly stimulated IgG responses and induced S1-specific IgA antibodies in serum. However, further investigation is required to determine whether the levels of IgA could be further increased through an additional booster or via intranasal immunisation.

On a different note, our study did not detect IgA antibodies in the intestinal mucosa, suggesting that subcutaneous immunisation failed to induce mucosal IgA responses. This limitation may be addressed by changing the vaccination routes to intranasal or intrarectal application.

Germinal centre reactions are essential for the maturation of antibody affinity and were shown to be enhanced following VLP immunisation. We previously observed that these GC reactions were accelerated when TLR7 signalling was activated during VLP immunisation [33, 44]. Specifically, downstream MyD88 signalling—particularly B cell-intrinsic TLR7 signalling—played a role in GC reactions [45, 46].

In this study, the AP205-S1 construct, which contained TLR7 ligands, successfully induced high-affinity antibodies in mice. This suggests that the vaccine effectively stimulated S1-specific GC reactions, although these reactions were not directly examined. High-affinity antibodies have been linked to the capacity to neutralise viruses, as seen in HIV-1 patients. The extreme mutations in the Ig variable regions of these patients allowed for broad neutralisation of various HIV-1 strains [47].

Furthermore, repeated exposure to antigens has been shown to enhance antibody affinities in convalescent patients, leading to improved neutralising abilities against SARS-CoV-2 [48]. AP205-S1 was notably effective in this study, eliciting high-affinity antibodies after just one booster immunisation. It would be valuable to investigate whether an additional booster could further increase the level of high-affinity antibodies and result in longer-lasting antibody responses, as this aspect was not assessed in the current study.

After confirming that the mice produced high-affinity antibodies, we tested their neutralisation potential. Consistent with the significant levels of high-affinity antibodies observed in the sera on day 49, S1-specific antibodies demonstrated strong neutralisation capabilities. The ability to neutralise PEDV increased alongside rising antibody levels and showed a dramatic enhancement after the booster was administered. The CPE results reflected the overall neutralising effects of all types of antibodies present in the samples.

According to IFA results, IgG antibodies found in both serum and mucosal tissues exhibited viral neutralising functions. However, we did not determine whether IgA antibodies in the immunised sera could neutralise PEDV in our study. Other research has suggested that IgA antibodies can neutralise SARS-CoV-2 [49], and that high-avidity IgA antibodies bind specifically to surface antigens, which inhibits the division of enteropathogens—a phenomenon referred to as “enchained growth” [50]. Therefore, we hypothesise that the S1-specific IgA antibodies in the immunised sera may also contribute to viral neutralisation.

Mucosal IgA plays a crucial role in defending against PEDV. However, we did not detect IgA antibodies in the fecal supernatants because the immunisation route used was systemic. Previous studies have shown that intranasal administration of VLPs can induce mucosal IgA responses, while subcutaneous injections do not have this effect [41], which aligns with our findings. Additionally, we are assessing mucosal IgA antibody levels through intranasal immunisation, although these results are not included in the current manuscript. Our results demonstrate that AP205-S1 can effectively stimulate the production of respiratory and intestinal IgA antibodies via intranasal immunisation.

The ideal candidates for a PEDV vaccine should be able to induce broadly cross-reactive antibody responses, allowing a single vaccination to protect against various variants. In this study, we demonstrated that the AP205-S1 vaccine elicited antibodies that neutralised the parental PEDV strain (KB2013) and effectively neutralised the G-I strain AH2018-HF1. Interestingly, the neutralising titres against AH2018-HF1 were slightly higher than those against KB2013. These results suggest that AP205-S1 can induce cross-reactive neutralising antibodies in mice.

Finally, the piglets immunised with AP205-S1 produced S1-specific IgG antibodies after receiving two doses. These antibodies were able to recognise and neutralise PEDV viruses in vitro. As a result, the piglets experienced less severe diarrhoea, reduced viral shedding, and displayed more energetic activity. However, these findings did not reach statistical significance, likely due to the small sample size, which could be increased in future studies to improve results. Therefore, it is recommended that the vaccine be administered to pregnant sows to protect their newborns.

Observations in individuals infected with SARS-CoV-2 after vaccination indicate that viral shedding is not significantly affected. This may be attributed to the limited presence of specific IgA antibodies, which could be a common issue with coronavirus vaccines. In the case of newborn piglets, they rely on passive immunity from maternal antibodies instead of active immunisation. Due to the low permeability of the sow’s placenta, piglets are born agammaglobulinemic and must depend on lactogenic immunity. In this context, maternal immunity refers specifically to lactogenic immunity.

To our knowledge, no research has systematically studied lactogenic immune responses following VLP vaccination in mice. However, previous work by Lu et al. demonstrated that VLP vaccines incorporating B-cell epitopes of PEDV provided lactogenic immunity to neonatal piglets [37]. The efficacy of the PEDV vaccine should focus on its ability to induce maternal antibodies, which is the next area we plan to investigate further, including the study of lactogenic immunogenicity. Additionally, we may consider using younger piglets with stronger signs of disease as a challenge.

In summary, we presented a vaccine candidate for PEDV, named AP205-S1, which is based on VLP. This vaccine was created through a specific covalent bond between SpyCatcher and SpyTag. AP205-S1 retained the viral nanoparticle structure and packaged ssRNA, resulting in a strong immune response when administered to mice. High titres of IgG antibodies were produced systemically, which could completely neutralise PEDV viruses and prevent them from infecting Vero cells in vitro. Importantly, these antibodies also cross-neutralised other strains of PEDV, indicating that AP205-S1 elicited cross-reactive antibody responses in mice. Additionally, the vaccine generated specific IgG responses in piglets, effectively neutralising PEDV in vitro. Although the clinical signs in immunised pigs were reduced, the difference was not statistically significant. This vaccine has considerable potential for further development, presenting an appealing alternative for preventing PEDV.

## Supplementary Information

Additional file: **1 Sequence alignment of S1 proteins of AH2018-HF1 and PEDV-KB2013 strains.**

## Data Availability

All data generated or analysed during this study are included in this published article.
