# Supplementary material for: A bacteriophage-based virus-like particle vaccine induces cross-reactive neutralising antibodies against porcine epidemic diarrhoea viruses (PEDV)
Source: Vet Res. 2025 Jul 1;56:128. doi: 10.1186/s13567-025-01559-z (PMC12210915; doi:10.1186/s13567-025-01559-z)
Supplement: Supplementary file 1 — Additional file: 1 Sequence alignment of S1 proteins of AH2018-HF1 and PEDV-KB2013 strains. [file 13567_2025_1559_MOESM1_ESM.pdf]

```
              1        10        20        30        40        50        60        70        80        90        100
AH2018-HF1    MTPLIYFWLFLPVLLTLSLPQDVTRCQSTINFRRFFSKFNVQAPAVVVLGGYLPSMN....SSSWYCGTGIETDSGVHGIFLSYIDSGQGFEIGISQEPFDPSGYQLYLH
PEDV-KB2013   MKSLTYFWLFLPVLSTLSLPQDVTRCSANTNFRRFFSKFNVQAPAVVVLGGYLPIGENQGVNSTWYCAGQHPTASGVHGIFVSHIRGGHGFEIGISQEPFDSSGYQLYLH

                110       120       130       140       150       160       170       180       190       200       210
AH2018-HF1    KATNGNTSAIARLRICQFPDNKTLGPTV.NDVTTGRNCLFNKAIPALQDGKNIVVGITWDNDRVTVFADKIYHFYIKNDWSRVATRCYNKRSCAMQYVYTPTYYMLNVTS
PEDV-KB2013   KATNGNTNATARLRICQFPSIKTLGPTANNDVTTGRNCLFNKDIPA.HMSEHSVVGITWDNDRVTVFSDKIYYFYFKNDWSRVATKCYNSGGCAMQYVYEPTYYMLNVTS

                220       230       240       250       260       270       280       290       300       310       320
AH2018-HF1    AGEDGIYYEPCTANCSGYAANVFATDSNGHIPEGFSFNNWFLLSNDSTLLHGKVVSNQPLLVNCLWAIPKIYGLGQFFSFNQTMDGVCNGAAAQRAPEALRFNINDTFVI
PEDV-KB2013   AGEDGISYQLCTANCIGYAANVFATEPNGHIPEGFSFNNWFLLSNDSTLVHGKVVSNQPLLVNCLLAIPKIYGLGQFFSFNQTIDGVCNGAALQRAPEALRFNINDTSVI

                330       340       350       360       370       380       390       400       410       420       430
AH2018-HF1    LAEGSIVLHTALGTNLSFVCSNSSDPHKAIFTIPLGVTEVPYYCFLKVDTYKSTVYKFLAVLPPTVKEIVITKYGDVYVNGFGYLHLGLLDAVTINFTGHGTDDDVSGFW
PEDV-KB2013   LAEGSIVLHTALGTNLSFVCSNSSDPHLATFAMPLGATQVPYYCFLKVDTYNSTVYKFLAVLPPTVREIVITKYGDFYVNGFGYLHLGLLDAVTINFTGHGTDDDVSGFW

                440       450       460       470       480       490       500       510       520       530       540
AH2018-HF1    TVASTNFVDALIEVQGTAIQRILYCDDPVSQLKCSQVSFDLDDGFYPISSRNLLSHEQPISFVTLPSFNDHSFVNITVSAAFGGHSGANLIASDTTINGFSSFCVDTRQF
PEDV-KB2013   TIASTNFVDALIEVQGTAIQRILYCDDPVSQLKCSQVAFDLDDGFYPISSRNLLSHEQPISFVTLPSFNDHSFVNITVSASFGGHSGANLIASDTTINGFSSFCVDTRQF

                550       560       570       580       590       600       610       620       630       640       650
AH2018-HF1    TITLFYNVTNSYGYVSKSQDSNCPFTLQSVNDYLSFSKFCVSTSLLAGACTIDLFGYPEFGSGVKFTSLYFQFTKGELITGTPKPLQGVTDVSFMTLDVCTKYTIYGFKG
PEDV-KB2013   TISLFYNVTNSYGYVSKSQDSNCPFTLQSVNDYLSFSKFCVSTSLLASACTIDLFGYPEFGSGVKFTSLYFQFTKGELITGTPTPLEGVTDVSFMTLDVCTQYTIYGFKG

                660       670       680       690       700       710       720
AH2018-HF1    EGIITLTNSSFLAGVYYTSDSGQLLAFKNVTSGAVYSVTPCSFSEQAVYVDDDIVGVISSLSNSTFNNTR
PEDV-KB2013   EGIITLTNSSFLAGVYYTSDSGQLLAFKNVISGAVYSVTPCSFSEQAAYVDGDIVGVISSLSSSTFNSTR
```
